# Supplementary material for: Empirical methods for controlling false positives and estimating confidence in ChIP-Seq peaks
Source: BMC Bioinformatics. 2008 Dec 5;9:523. doi: 10.1186/1471-2105-9-523 (PMC2628906; doi:10.1186/1471-2105-9-523)
Supplement: Additional File 1 — A variety of html documents from the USeq web site detailing the available applications, their best usage, output file type descriptions, command line menus, etc. [file 1471-2105-9-523-S1.zip › USeqUserGuides/JavaDoc.zip!/JavaDoc/index-files/index-10.html]

J-Index


|  |  |  |
| --- | --- | --- |
| **Overview** \| Package \| Class \| Use \| **Tree** \| **Deprecated** \| **Index** \| **Help** | |  |
| **PREV LETTER**   **NEXT LETTER** | **FRAMES**    **NO FRAMES**     **All Classes** |  |


A B C D E F G H I J L M N O P Q R S T U V W X Z 

---


## **J**

**Job** - Class in util.gen: **Job(String[])** - Constructor for class util.gen.Job: **JohnsonToTag** - Class in edu.utah.seq.parsers: **JohnsonToTag()** - Constructor for class edu.utah.seq.parsers.JohnsonToTag: **joinSegments(ArrayList)** - Static method in class gata.main.GATAUtil: Takes an ArrayList of int[2]'s representing start stop coords kills the dups, and those cotained within another seg, will merge overlapping segments. **joinSegments(ArrayList)** - Static method in class util.bio.annotation.GeneGroup: Takes an ArrayList of int[2]'s representing start stop coords kills the dups, and those cotained within another seg, will merge overlapping segments. **JQSub** - Class in util.apps: Fires command lines on clusters through qsub. **JQSub(String[])** - Constructor for class util.apps.JQSub

---


|  |  |  |
| --- | --- | --- |
| **Overview** \| Package \| Class \| Use \| **Tree** \| **Deprecated** \| **Index** \| **Help** | |  |
| **PREV LETTER**   **NEXT LETTER** | **FRAMES**    **NO FRAMES**     **All Classes** |  |


A B C D E F G H I J L M N O P Q R S T U V W X Z 

---
